# Exposure of a Microvascular Anastomotic Coupler in Head and Neck Reconstruction: A Case Report

**Published:** 2014-04-09

**Authors:** Tateki Kubo, Natsuko Kitamura, Motohiro Onoda, Daisuke Maeda, Ken Matsuda, Ko Hosokawa

**Affiliations:** ^a^Department of Plastic Surgery, Osaka University Graduate School of Medicine; ^b^Department of Plastic Surgery, Osaka Rosai Hospital, Osaka, Japan

**Keywords:** microvascular anastomotic coupler, venous anastomosis, complications, exposure, head and neck reconstruction

## Abstract

**Objective:** We present a case of exposure of a microvascular anastomotic coupler. **Methods:** We performed venous anastomoses using microvascular anastomotic couplers in head and neck reconstruction. **Results:** A microvascular anastomotic coupler was exposed in the seventh month postoperation, and an anastomosed vein was disrupted. **Conclusions:** Although the usefulness of the microvascular anastomotic coupler in microsurgical flap reconstruction is not in doubt, as described previously, we believe that it is necessary to remember that use of a microvascular anastomotic coupler involves potential risks of exposure and anastomosed vessel disruption.

Along with the recent progress in microsurgery, free flap transfer has reached the stage where it can be safely utilized clinically as a reconstructive procedure, with success rates exceeding 95%.^1^ Improvement in devices for microsurgery, such as, operating microscopes, microsurgical instruments, and microvascular sutures, have contributed significantly to the success. However, a handsewn anastomosis is still a technically demanding procedure, particularly when dealing with veins. The concept of the microvascular anastomotic coupler, consisting of 2 metal rings with 12 interlocking pins and holes, was first introduced in 1962,^2^ and it was adapted in 1986 by Ostrup and Berggren into the current Synovis microvascular anastomotic device (GEM coupler, Synovis Micro Companies Alliance, Birmingham, Alabama).^3^ The microvascular anastomotic coupler is time-saving and far easier to use as compared with a handsewn venous anastomosis.^4^ Since then, the microvascular anastomotic coupler has been an alternative to the handsewn process for venous anastomosis^4^ and has achieved a low venous thrombosis rate, 1.7% in a total of 3497 venous anastomoses.^5^ Despite such a high success rate using the microvascular anastomotic coupler, a few late complications when applied in the extremities, foreign body sensation and migration, have been reported.^6^^-^8 Here, we report a case of late exposure of a microvascular anastomotic coupler after use for head and neck reconstruction.

## CASE REPORTS

**Figure 1:**
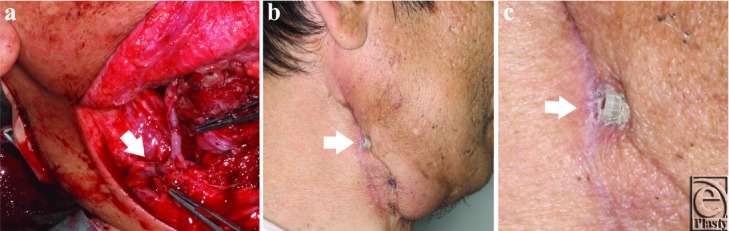
(a) Intraoperative view of the microvascular anastomotic coupler used to join the concomitant vein with the external jugular vein (arrow). (b) Exposure of the coupler near the suture line on the patient's neck in the 7 month postoperation (arrow). (c) More than half of the rings of the coupler were exposed (arrow).

Right hemiglossectomy and ipsilateral supraomohyoid neck dissection were performed in a 53-year-old man with tongue cancer. The defect of the tongue was covered with a free anterolateral thigh flap, and the transverse branch of the lateral circumflex femoral artery was anastomosed to the facial artery with a handsewn suture. Two venous anastomoses were performed to join concomitant veins with the external jugular and anterior jugular vein using 2.5 mm microvascular anastomotic couplers (Fig 1a). The flap survived completely, and the patient underwent postoperative radiation therapy with a total dose of 60 grays. Seven months after surgery, although the transferred flap appeared healthy, the patient visited us because of exposure of the microvascular anastomotic coupler near the suture line on the right side of his neck (Fig 1b). More than half of the rings of the coupler, which seemed to be the one used for anastomosis to the external jugular vein on the sternocleidomastoid muscle, were exposed (Figs 1a, c). The apposition of the interlocking pins and rings was maintained, but the anastomosed vein could not be seen in the rings of the coupler, which meant the anastomosed vein had been disrupted. We then removed the coupler by pulling it manually, and the wound healed without any problems.

## DISCUSSION

The microvascular anastomotic coupler has been used for microvascular breast, head, and neck and extremity reconstruction. It can be applied for both arterial and venous anastomosis, but use for veins has been well established. Recently, Grewal et al^5^ performed a systematic review of the literature to examine the utility of the microvascular anastomotic coupler in free tissue transfer and revealed that only 61 (1.7%) venous thromboses occurred in 3497 venous anastomoses and that 12 (3.6%) occurred in 342 arterial anastomoses. They concluded that the microvascular anastomotic coupler achieved results superior to those of a sutured anastomosis when used by experienced surgeons. On the contrary, late complications have been reported when the microvascular anastomotic coupler has been used in the extremities.^6^^-^8 Three cases of foreign body sensation on the dorsum of the hand^6^^,^^7^ and 1 case of migration on the dorsum of the foot^8^ have been reported, probably because of the nature of the relatively thin soft tissue in those areas. Eventually, it was necessary to surgically remove the coupler in all cases. In contrast, to the best of our knowledge, there have been no such reports of late complications when the microvascular anastomotic coupler has been used in breast or head and neck reconstruction. There is an article mentioning microvascular anastomotic coupler exposure in the face, but this case occurred after the patient developed a suture line breakdown overlying the coupler in the relatively early postoperative period (in the sixth week postoperation).^9^ We consider that there is lower probability for late complications when using the microvascular anastomotic coupler due to the thickness of the soft tissue in the breast or head and neck being sufficient to cover the coupler. In our case, the microvascular anastomotic coupler was exposed 7 months after head and neck reconstruction. It was located near the suture line on the sternocleidomastoid muscle. We speculate that the reason why the coupler was exposed was because it was placed near the suture line, which is mechanically weak, and the contraction of sternocleidomastoid muscle might have caused shear stress in the skin through contact with the coupler. In addition, the patient underwent postoperative radiation therapy.

In our case, the apposition of the interlocking pins and rings was maintained, but the anastomosed vein was not present within the rings of the coupler. Therefore, the anastomosed vein seemed to have already been disrupted. Since 2 venous anastomoses were performed in our case and the other vein was patent, the transferred flap remained viable without any compromise. However, disruption of a vascular anastomosis in free flap reconstruction has the potential for severe complications including bleeding, flap congestion, and ischemia.^8^ Generally, in the late postoperative period, neovascularization from the recipient bed into the flap occurs and this could be sufficient to maintain flap viability independent of the primary anastomosed vessels. However, the extent of neovascularization depends on many variable factors, such as, the volume of transferred tissue, type of flap, and capacity for neovascularization of the recipient bed,^10^ and in fact, Fisher and Wood^11^ and Sadove and Kanter^12^ have described cases of complete loss of a free flap 7 and 3 months, respectively, after transfer when the pedicle supply was disrupted. Therefore, although the usefulness of the microvascular anastomotic coupler in microsurgical flap reconstruction is not in doubt, as described previously, we believe that plastic surgeons need to remember that use of the microvascular anastomotic coupler involves potential risks of exposure and disruption of anastomosed vessels.

## CONCLUSIONS

We reported our experience of late exposure of a microvascular anastomotic coupler in head and neck reconstruction. Therefore, although the usefulness of the microvascular anastomotic coupler in microsurgical flap reconstruction is not in doubt, as described previously, we believe that plastic surgeons need to remember that use of the microvascular anastomotic coupler involves potential risks of exposure and disruption of anastomosed vessels.
